# The invasive Korean bush mosquito *Aedes koreicus* (Diptera: Culicidae) in Germany as of 2020

**DOI:** 10.1186/s13071-021-05077-7

**Published:** 2021-11-12

**Authors:** Nicolas Hohmeister, Doreen Werner, Helge Kampen

**Affiliations:** 1 Friedrich-Loeffler-Institut, Federal Research Institute for Animal Health, Insel Riems, Greifswald, Germany; 2 Leibniz-Centre for Agricultural Landscape Research, Muencheberg, Germany

**Keywords:** *Aedes japonicus*, Cemetery, Distribution, Genetic identification, *Hulecoeteomyia koreica*, Invasive mosquito species, Germany, Mueckenatlas

## Abstract

**Background:**

The Korean bush mosquito *Aedes koreicus* was recently reported to have established a population in western Germany (Wiesbaden) in 2016. The species is difficult to distinguish morphologically from its close relative, the invasive Japanese bush mosquito *Ae. japonicus*, which is already widely distributed in many parts of Germany, including the area colonised by *Ae. koreicus*. Genetic confirmation of morphologically identified “*Ae. japonicus*” collection material, however, had only been done exceptionally before the German *Ae. koreicus* population became known.

**Methods:**

Dried archived “*Ae. japonicus*” specimens both from the municipality of Wiesbaden and from deliberately and randomly selected distribution sites all over Germany were re-examined morphologically and genetically for admixture by *Ae. koreicus*. Moreover, cemeteries in the greater Wiesbaden area were sampled in 2019 and 2020 to check for *Ae. koreicus* spread. Korean and Japanese bush mosquitoes submitted to the German citizen science mosquito monitoring scheme “Mueckenatlas” in 2019 and 2020 were also subjected to particularly thorough species identification. The ND4 DNA sequences generated in this study in the context of species identification were phylogenetically compared to respective GenBank entries of *Ae. koreicus*. As a by-product, several genetic markers were evaluated for their suitability to identify *Ae. koreicus*.

**Results:**

*Aedes koreicus* specimens could be identified in mosquito collection material and submissions from Wiesbaden from 2015 onwards, suggesting establishment to have happened in the same year as *Ae. japonicus* establishment. Detections of *Ae. koreicus* from 2019 and 2020 in Wiesbaden indicate a negligible enlargement of the populated area as described for 2018. Two *Ae. koreicus* specimens were also submitted from the city of Munich, southern Germany, in 2019 but further specimens could not be identified during immediate local inspections. Comparison of ND4 sequences generated in this and other studies demonstrate a high degree of homology, suggesting that this DNA region is not informative enough for clarification of origins and relationships of *Ae. koreicus* populations. For genetic identification of *Ae. koreicus*, PCR primers used for classical CO1 barcoding were found to lead to mismatches and produce no or incorrect amplicons. Alternative CO1 primers or a validated ND4 marker should be used instead.

**Conclusions:**

*Aedes koreicus* is probably introduced into Germany every now and then but rarely succeeds in becoming established. As with most European populations, the German population is characterised by a limited expansion tendency. Since *Ae. koreicus* is a potential vector, however, Asian bush mosquitoes found at new places should be examined quite carefully and known distribution areas of *Ae. japonicus* regularly checked for the presence of *Ae. koreicus*.

**Graphical Abstract:**

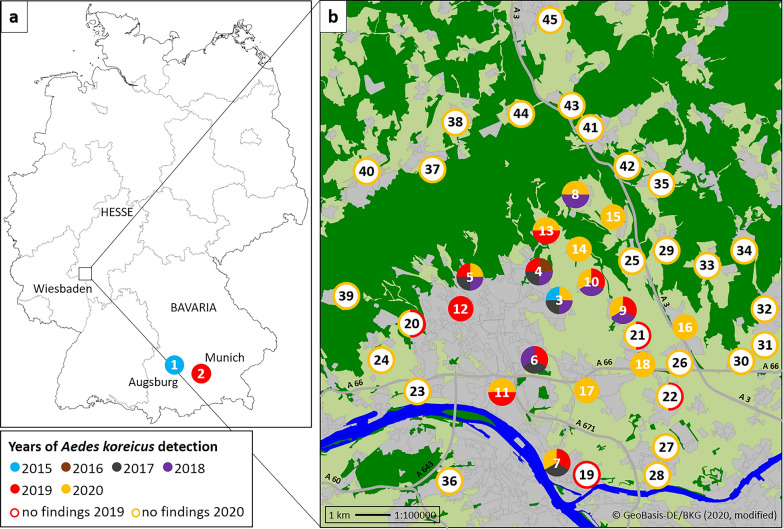

## Background

The Korean bush mosquito, *Aedes* (*Hulecoeteomyia*) *koreicus* (Edwards, 1917), has its native distribution area in Japan, Korea, northern China and southern Russia [1, 2]. Following *Ae. albopictus, Ae. aegypti* and *Ae. japonicus* [3], it became invasive to Europe in 2008 when the first specimens were reported from Belgium [4]. In 2011, the species emerged in Italy, in 2013 in Russia (eastern Black Sea coast), Switzerland and Slovenia, in 2015 in Germany, in 2016 in Hungary and in 2018 in Austria [5–11]. In contrast to Italy, where the species spread after establishment [12, 13], no significant dispersal has been reported from other colonised areas, while the detection of a single specimen in Germany in 2015 was apparently not even linked to population development [14].

Little is known about the vector potential of *Ae. koreicus*. It is stated to be a vector of Japanese encephalitis virus in Russia [1, 15] and has proven able to transmit *Dirofilaria immitis* [16, 17] and *Brugia malayi* [18] in the laboratory. Vector competence for chikungunya virus, although low, was also reported [19].

*Aedes koreicus* is closely related and both morphologically and genetically very similar to the Japanese bush mosquito *Ae. japonicus.* Genetic analysis, though, is believed to unambiguously lead to reliable distinction in all life stages while the species can be easily confused morphologically: major characteristics (dark subbasal band on the hind femur in *Ae. japonicus* but not in *Ae. koreicus* and a pale basal band on hind tarsomere 4 in *Ae. koreicus* but absent in *Ae. japonicus* [9, 20]) may be inarticulate and overlooked. Taxonomically, both species belong to the Japonicus Group of the *Aedes* subgenus *Finlaya* [2]. In fact, *Ae. koreicus* was once considered an *Ae. japonicus* variant [1], and—based on genetic data—Cameron et al. [21] have suggested its reclassification as a subspecies of *Ae. japonicus*. *Aedes japonicus* is another invasive mosquito species originating from Asia, which was first detected in Europe in 2000 [22]. From 2003 onwards, it has spread into numerous European countries and over considerable parts of southern and northwestern Germany [23–25].

Following the submission of an *Ae. koreicus* female from the South German federal state of Bavaria in 2015 [9], a single larval specimen of this species was found in 2016 in a cemetery in the municipality of Wiesbaden, federal state of Hesse, western Germany, triggering further investigations in 2017 and 2018 during which local establishment of *Ae. koreicus* was demonstrated [20, 26, 27].

Before the background that *Ae. japonicus* had been found in the Wiesbaden region for the first time in 2015 and continuously and widely distributed afterwards [24, 25, 28], we thoroughly re-analysed our “*Ae. japonicus*” collection material from the presently known *Ae. koreicus* population area in Wiesbaden and several other randomly selected collection sites in Germany, similar to Slovenian investigations in 2016 [10]. We also present *Ae. koreicus* findings from 2019 and 2020 in Germany and discuss the genetic identification of this species.

## Methods

### Study material

Mosquitoes analysed included *Ae. japonicus*/*koreicus*-like specimens and were derived from larval field collections and submissions of adults to the citizen science project “Mueckenatlas” from 2019 and 2020 as well as from archived mosquito material (adults prepared from larval samplings and Mueckenatlas submissions) collected from 2015 to 2018.

**Fig. 1:**
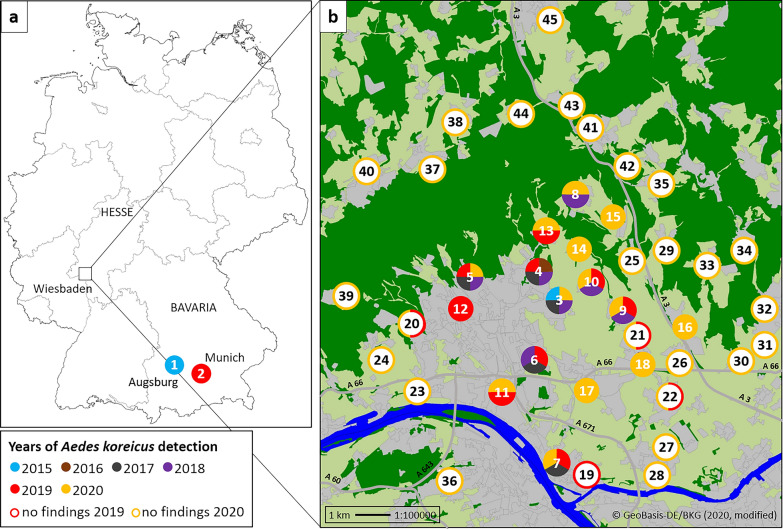
*Aedes koreicus* collection sites in Germany, based on Werner et al. [9], Pfitzner et al. [19, 25], Steinbrink et al. [26] and the present study, as well as sampling sites found negative for *Ae. koreicus* in this study (see Table 1 for site details). **a** Overview of Germany with federal states and cities named where *Ae. koreicus* specimens were found. **b** Detail enlargement of the greater Wiesbaden area where the *Ae. koreicus* population occurs.

Based on Vezzani et al. [29], field sampling in search of *Ae. koreicus* in the Wiesbaden area was conducted in cemeteries from 27 to 29 August 2019, and from 11 to 15 August and 12 to 16 September 2020, with sampling site selection according to previous *Ae. koreicus* detections in that area. In 2019, 12 sites were sampled, including five previously positive, five previously negative and two never sampled before (Fig. 1b, Table 1). In 2020, 17 sites were inspected, including five positive in previous studies, eight previously negative, one new in this study but already checked in 2019, and three checked for the first time (Fig. 1b, Table 1). An additional 21 sites were examined outside the Wiesbaden municipality in 2020, eight of which had been inspected previously by Pfitzner et al. [20, 26], while 13 were checked for the first time (Fig. 1b, Table 1).

**Table 1:** Details of sites sampled for *Ae. koreicus* in the municipality of Wiesbaden and the cities of Augsburg and Munich (site numbers refer to Fig. 1)

Site No.	Location	Habitat	*Ae. koreicus*/*Ae. japonicus*	Mode of collection	Month(s)/year(s) of collection	References
1	Augsburg	Urban area	+/−	MA submission	Jun 2015	[9]
2	Munich	Urban area	+/−	2 MA submissions	Sep 2019	This study
3	Wiesbaden-Bierstadt	Urban area	+/−	2 MA submissions^a^	Aug 2015	This study
			+/−	MA submission^a^	Aug 2017	This study
		Cemetery, field/garden	+/−	Ovitrapping	Aug, Oct 2017	[20, 26]
		urban area	+/nd	BG-Sentinel trapping	Aug–Oct 2017	[27]
			+/nd	Dipping/sieving	May, July 2018	
		Cemetery	+/+	Dipping/sieving^a^	Aug 2018	This study
			+/−		Sep 2018	
			−/−	Dipping/sieving	Aug 2019	This study
			+/−		Aug 2020	
4	Wiesbaden-Sonnenberg	Cemetery	+/−	Dipping/sieving	Sep 2016	[20, 26]
			+/+	Ovitrapping, dipping/sieving	Aug, Oct 2017	[20, 26]
			+/−	Dipping/sieving	2018	
			+/−		Aug 2019	This study
		Forest	+/−	Ovitrapping	Jul–Oct 2017	[20]
		Industrial area	+/−			
5	Wiesbaden-Northeast	Cemetery	+/−	Dipping/sieving	Sep 2017	[20, 26]
			+/+		2018	
			+/+		Aug 2019	This study
			+/+		Aug 2020	
6	Wiesbaden-Southeast	Cemetery	+/+	Dipping/sieving	Sep 2017	[20, 26]
			+/+		2018	
			+/+		Aug 2019	This study
7	Mainz-Kastel	Cemetery	+/−	Dipping/sieving	Sep 2017	[20, 26]
			+/−		Aug 2019	This study
			+/−		Aug 2020	
8	Wiesbaden-Naurod	Cemetery	+/−	Dipping/sieving	2018	[20, 26]
			+/−		Aug 2020	This study
9	Wiesbaden-Igstadt	Cemetery	+/+	Dipping/sieving	2018	[20, 26]
		Rural area	+/−	MA submission	Oct 2019	This study
		Cemetery	+/+	Dipping/sieving	Aug 2020	
10	Wiesbaden-Kloppenheim	Rural area	+/−	MA submission	Jul 2018	This study
		Cemetery	+/−	Dipping/sieving	Aug 2019	
		Rural area	+/−	3 MA submissions	Jun 2020	
11	Wiesbaden-Biebrich	Urban area	+/−	MA submission	May 2019	This study
		Cemetery	+/−	Dipping/sieving	Aug 2019	
			+/−		Sep 2020	
12	Wiesbaden-Klarenthal	Urban area	+/−	MA submission	Jul 2019	This study
13	Wiesbaden-Rambach	Cemetery	+/+	Dipping/sieving	Aug 2019	This study
			+/+		Aug 2020	
14	Wiesbaden-Hessloch	Cemetery	+/−	Dipping/sieving	Sep 2020	This study
15	Wiesbaden-Auringen	Cemetery	+/+	Dipping/sieving	Sep 2020	This study
16	Wiesbaden-Breckenheim	Cemetery	+/−	Dipping/sieving	Aug 2020	This study
17	Wiesbaden-Erbenheim	Cemetery	+/−	Dipping/sieving	Aug 2020	This study
18	Wiesbaden-Nordenstadt	Rural area	+/−	MA submission	Oct 2020	This study
19	Mainz-Kostheim	Cemetery	−/−	Dipping/sieving	Aug 2019	This study
20	Wiesbaden-Dotzheim	Cemetery	−/+	Dipping/sieving	Aug 2019	This study
					Aug, Sep 2020	
21	Wiesbaden-Nordenstadt	Cemetery	−/−	Dipping/sieving	Aug 2019	This study
					Aug, Sep 2020	
22	Wiesbaden-Delkenheim	Cemetery	−/−	Dipping/sieving	Aug 2019	This study
					Aug, Sep 2020	
23	Wiesbaden-Schierstein	cemetery	−/−	Dipping/sieving	Aug, Sep 2020	This study
24	Wiesbaden-Frauenstein	Cemetery	−/+	Dipping/sieving	Aug, Sep 2020	This study
25	Wiesbaden-Medenbach	Cemetery	−/−	Dipping/sieving	Aug, Sep 2020	This study
26	Hofheim-Wallau	Cemetery	−/−	Dipping/sieving	Aug, Sep 2020	This study
27	Hochheim—new cemetery	Cemetery	−/−	Dipping/sieving	Aug, Sep 2020	This study
28	Hochheim—old cemetery	Cemetery	−/+	Dipping/sieving	Aug, Sep 2020	This study
29	Hofheim-Wildsachsen	Cemetery	−/+	Dipping/sieving	Aug, Sep 2020	This study
30	Hofheim-Diedenbergen	Cemetery	−/−	Dipping/sieving	Aug, Sep 2020	This study
31	Hofheim-Marxheim	Cemetery	−/+	Dipping/sieving	Aug, Sep 2020	This study
32	Hofheim	Cemetery	−/+	Dipping/sieving	Aug, Sep 2020	This study
33	Hofheim-Langenhain	Cemetery	−/−	Dipping/sieving	Aug, Sep 2020	This study
34	Hofheim-Lorsbach	cemetery	−/+	Dipping/sieving	Aug, Sep 2020	This study
35	Eppstein-Bremthal	Cemetery	−/−	Dipping/sieving	Aug, Sep 2020	This study
36	Mainz-Gonsenheim	Cemetery	−/+	Dipping/sieving	Aug, Sep 2020	This study
37	Taunusstein-Wehen	Cemetery	−/−	Dipping/sieving	Aug, Sep 2020	This study
38	Taunusstein-Neuhof	Cemetery	−/−	Dipping/sieving	Aug, Sep 2020	This study
39	Schlangenbad-Georgenborn	Cemetery	−/+	Dipping/sieving	Aug, Sep 2020	This study
40	Taunusstein-Hahn	Cemetery	−/+	Dipping/sieving	Aug, Sep 2020	This study
40	Taunusstein-Bleidenstadt	Cemetery	−/−	Dipping/sieving	Aug, Sep 2020	This study
41	Niedernhausen-Königshofen	Cemetery	−/−	Dipping/sieving	Aug, Sep 2020	This study
42	Niedernhausen	Cemetery	−/+	Dipping/sieving	Aug, Sep 2020	This study
43	Niedernhausen-Niederseelbach	Cemetery	−/−	Dipping/sieving	Aug, Sep 2020	This study
44	Niedernhausen-Engenhahn	Cemetery	−/+	Dipping/sieving	Aug, Sep 2020	This study
45	Idstein	Cemetery	−/−	Dipping/sieving	Aug, Sep 2020	This study

^a^Previously misidentified as *Ae. japonicus*; MA = Mueckenatlas, nd = no data

Examined cemeteries had different sizes and structures and were located in urban and rural and close to forested areas. Potential *Ae. koreicus* breeding sites were generally evenly distributed over the whole cemetery areas, with all detected ones being inspected during a visit.

Samples were taken by sieving water from flower vases, flower pot saucers and water bowls, and by dipping wells. Second to fourth bush mosquito larval instars as identified on the spot with the naked eye [30] were transferred into closable glass jars filled with water from the collection site and transported to the laboratory. There they were kept in the same jars at 24 °C, 70% RH and a 12 h light/12 h darkness regime until adult emergence, with the container lid replaced by a collection net. Until pupation, larvae were fed ground fish food (Tetra TabiMin, Melle, Germany) which was carefully added onto the water of the rearing containers once per day until spreading by the surface tension came to a standstill. As soon as adult emergence started, collection nets were removed daily and exposed to overnight freezing at −20 °C to kill the adults while being replaced on the rearing containers. Killed adults were stored frozen or pinned at room temperature.

In addition, Asian bush mosquitoes submitted to the Mueckenatlas in 2019 and 2020 were subjected to thorough species identification, and adult voucher specimens from our entomological collection (derived from both past Mueckenatlas submissions and field sampling), available in a dried form either in vials or pinned, were re-analysed. Particular attention was paid to archived specimens originating from the Wiesbaden area and specimens collected along a direct imaginary line from Augsburg to Wiesbaden, but randomly selected samples from other German “*Ae. japonicus*” distribution areas represented in the entomological collection were also checked.

### Species identification

Morphological species identification concentrated on females where distinguishing characters are more prominent than in males, using the keys provided by Tanaka et al. [2] and Pfitzner et al. [20].

Females morphologically pre-identified as *Ae. koreicus* or with ambiguous morphological characteristics were genetically identified by sequencing of polymerases chain reaction (PCR)-amplified DNA marker regions. For this purpose, total DNA was extracted from a single leg of each mosquito using the QIAamp DNA Mini Kit (Qiagen, Hilden, Germany) according to the manufacturer’s instructions. The extracted DNA was stored at −20 °C until further processing.

PCR amplification was comparatively performed on two genetic markers: the mitochondrial ND4 (nicotinamide adenine dinucleotide dehydrogenase subunit 4) gene and the CO1 (cytochrome oxidase c subunit 1) gene, with four different primer pairs targeting the latter. PCR protocols and primers were used as described by Kumar et al. [31], Lehmann et al. [32] and Zielke et al. [33].

Following agarose gel electrophoresis, PCR products were extracted by means of the QIAquick Gel Extraction Kit (Qiagen) and cycled unidirectionally using the PCR primers and the BigDye Terminator v3.1 Cycle Sequencing Kit (Thermo Fisher, Darmstadt, Germany) according to the manufacturer’s instructions, purified by NucleoSEQ^®^ columns (Macherey–Nagel, Düren, Germany) and sequenced on a 3500 Genetic Analyzer (Applied Biosystems/Hitachi, Darmstadt, Germany). Selected samples intended for deposition in GenBank were sequenced bidirectionally.

The Geneious Prime programme (version 2019.2.3, Biomatters, Ltd., Auckland, New Zealand) was used for sequence editing and alignments. Genetic data were checked against GenBank (https://blast.ncbi.nlm.nih.gov/Blast.cgi) and/or BOLD databases (https://www.boldsystems.org/).

### Phylogenetic analyses

ND4 sequences obtained for *Ae. koreicus* were compared to all corresponding sequences available in GenBank. To display genetic relationships between specimens or populations, a phylogenetic tree was calculated using the maximum likelihood method with evolutionary distances computed by the Tamura 3-parameter model [34]. Evolutionary analyses were conducted in MEGA X [35] using *Ae. japonicus* (KF211480) as an outgroup.

## Results

### Mosquito field collections in 2019 and 2020

In 2019 and 2020, cemetery collections in the Wiesbaden area produced bush mosquito larvae developing to 184 *Ae. koreicus* (58 in 2019, 126 in 2020) and 250 *Ae. japonicus* females (33 in 2019, 217 in 2020), and to 231 male specimens of either of these two *Aedes* species (71 in 2019, 160 in 2020). Out of 42 cemeteries sampled in the two years (Table 1), 14 were positive for *Ae. koreicus*, including seven of these positive for the first time, two thereof already checked by Pfitzner et al. [20, 26] (Fig. 1b). Four of the positive cemeteries were slightly outside the previously described *Ae. koreicus* population area [20, 26].

In 13 cemeteries, only *Ae. japonicus* was found, while in six cemeteries both *Ae. koreicus* and *Ae. japonicus* were present. East of the municipality of Wiesbaden, in the direction of Frankfurt International Airport, *Ae. koreicus* aquatic stages could not be found in 10 cemeteries checked in 2020.

### Mueckenatlas submissions 2019 and 2020

Six *Ae. koreicus* specimens were submitted to the Mueckenatlas in 2019, with four of them from three different collection sites in the Wiesbaden area and two from a single collection site in Munich (Fig. 1). In 2020, four *Ae. koreicus* individuals were received from two sites in the Wiesbaden area (three from one and one from a second site; Fig. 1a). The 2019 and 2020 Mueckenatlas collection sites in Wiesbaden were different, and four of the six Wiesbaden collection sites linked to Mueckenatlas submissions matched geographically with sites found positive for *Ae. koreicus* larvae in the field (Fig. 1b).

### Re-examination of archived mosquito material

Two “*Ae. japonicus*” specimens collected in the Wiesbaden area in 2015 and submitted to the Mueckenatlas monitoring scheme (Fig. 1b, Table 1) turned out to be *Ae. koreicus* after genetic identification. In addition, two *Ae. koreicus* specimens submitted from that area in 2017 and 2018 (one specimen per year) were found to be misidentified as *Ae. japonicus* (Fig. 1a). Furthermore, a vast majority of *Ae. koreicus* individuals (65 of 66) were found in two re-analysed larval collections from the same cemetery in the Wiesbaden municipality from 2018 (Fig. 1b, Table 1).

No admixture by *Ae. koreicus* was found in Asian bush mosquito material collected between 2016 and 2020 from other sites around the Wiesbaden area (*n* = 8), from sites in and around Augsburg (2016–2020: 17 sites), where the first *Ae. koreicus* specimen ever found in Germany originated, from sites along an imaginary line from Augsburg to Wiesbaden (2016: 5 sites, 2017: 18 sites, 2018: 4 sites, 2019: 8 sites, 2020: 9 sites), or from sites arbitrarily selected throughout the German *Ae. japonicus* distribution area (4–8 different sites per years 2015–2018).

### Genetic identification

**Fig. 2:**
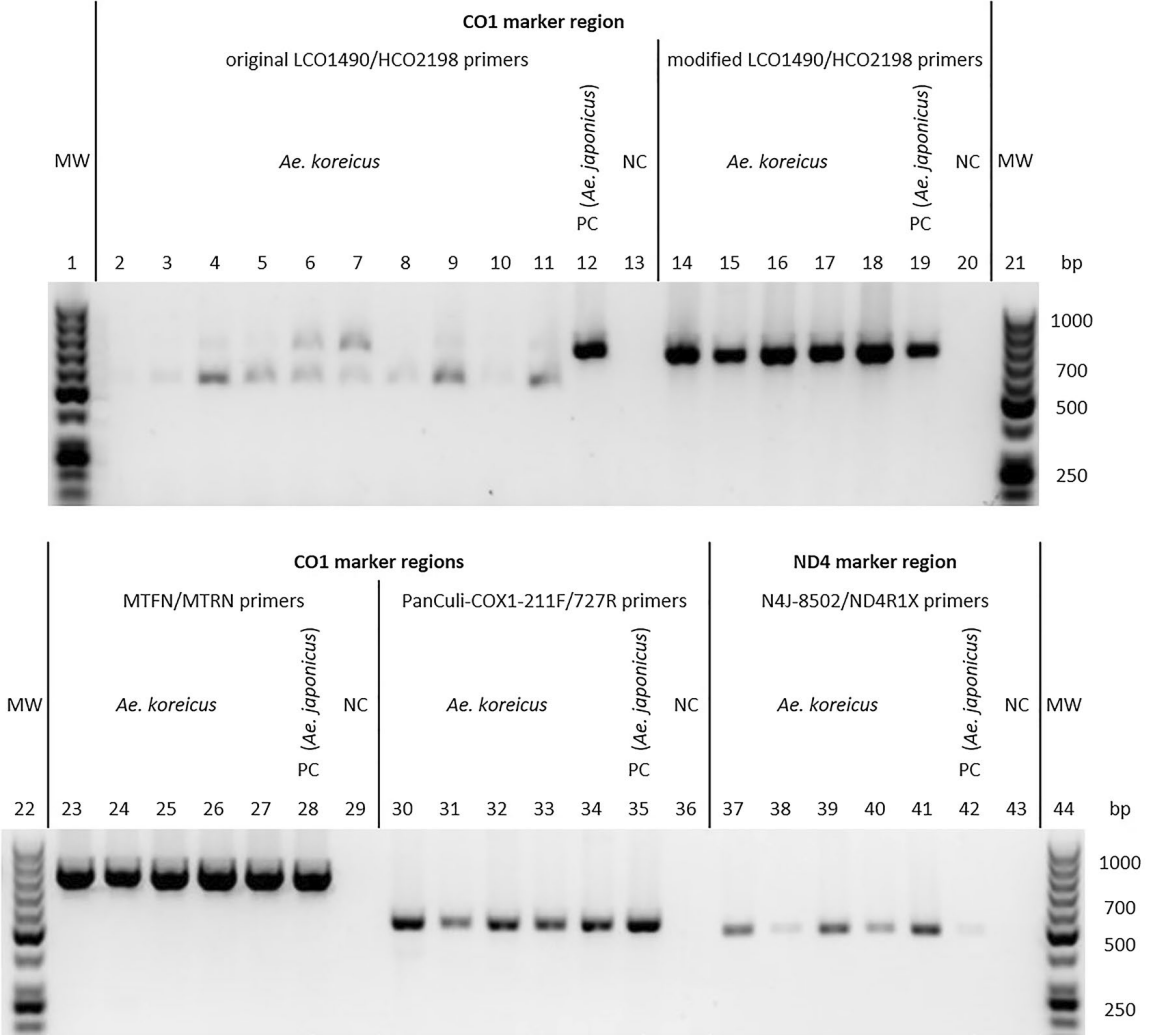
Image of ethidium bromide-stained agarose gels with *Ae. koreicus*-amplified DNA using original LCO1490/HCO2198 primers (lanes 2–11), modified LCO1490/HCO2198 primers (lanes 14–18), MTFN/MTRN primers (lanes 23–27), PanCuli-COX1-211F/Pan-Culi-COX1-727R primers (lanes 30–34) and N4J-8502/ND4R1X primers (lanes 37–41). PCRs with *Ae. japonicus*-DNA served as positive controls (PC; lanes 12, 19, 28, 35, 42), with ddH_2_O as negative controls (NC; lanes 13, 20, 29, 36, 43). MW = molecular weight marker (Thermo Fisher, Dreieich, Germany; lanes 1, 21, 22, 44)

PCR amplification of the CO1 marker region described by Hébert et al. [36] using the primers LCO1490 and HCO2198 [37] consistently produced incorrect or no amplicons from *Ae. koreicus* DNA. Often, gel electrophoresis revealed a shorter DNA fragment in addition to the expected one (ca. 710 base pairs [bp]) or the shorter fragment only (Fig. 2), with attempts to sequence them being unsuccessful. Alignment of the primers with two complete *Ae. koreicus* mitochondrial DNA sequences from GenBank (accession nos. NC_046946, MT093832) showed mismatches pertaining to two bases in the forward and four bases in the reverse primer, although not at the 3′-ends. PCRs with the same primers and lower annealing temperatures (50 and 52 °C instead of 54 °C) to compensate for the base mismatches did not improve the results. Newly constructed primers LCO1490mod (5′-TCTCAACAAATCATAAAGATATTGG-3′) and HCO2198mod (5′-TAAACTTCTGGGTGTCCGAAGAATCA-3′), 100% homologous to the annealing sites of the *Ae. koreicus* GenBank sequences, however, produced the expected PCR products (Fig. 2). These could be sequenced without difficulties, with sequences unambiguously identifying *Ae. koreicus*. A primer mixture consisting of equal concentrations of original and modified primers, and matching the total primer concentration according to the protocol, generated high-quality amplicons for both *Ae. koreicus* and 10 other arbitrarily selected Central European mosquito species (*Aedes caspius, Ae. japonicus, Ae. vexans, Ae. rossicus, Anopheles claviger, Culex hortensis, Cx. territans, Cx. torrentium, Culiseta annulata, Cs. longiareolata*; data not shown).

PCR amplifications using CO1-specific primer pairs according to Kumar et al. [31] (MTFN/MTRN) and Lehmann et al. [32] (PanCuli-COX1-211F/PanCuli-COX1-727R) and an ND4-specific primer pair according to Fonseca et al. [38] and Egizi and Fonseca [39] (N4J-8502/ND4R1X) also produced clear bands on the gel (Fig. 2), the corresponding DNA fragments of which could be sequenced and assigned to *Ae. koreicus* without problems.

### Phylogenetic analyses

**Fig. 3:**
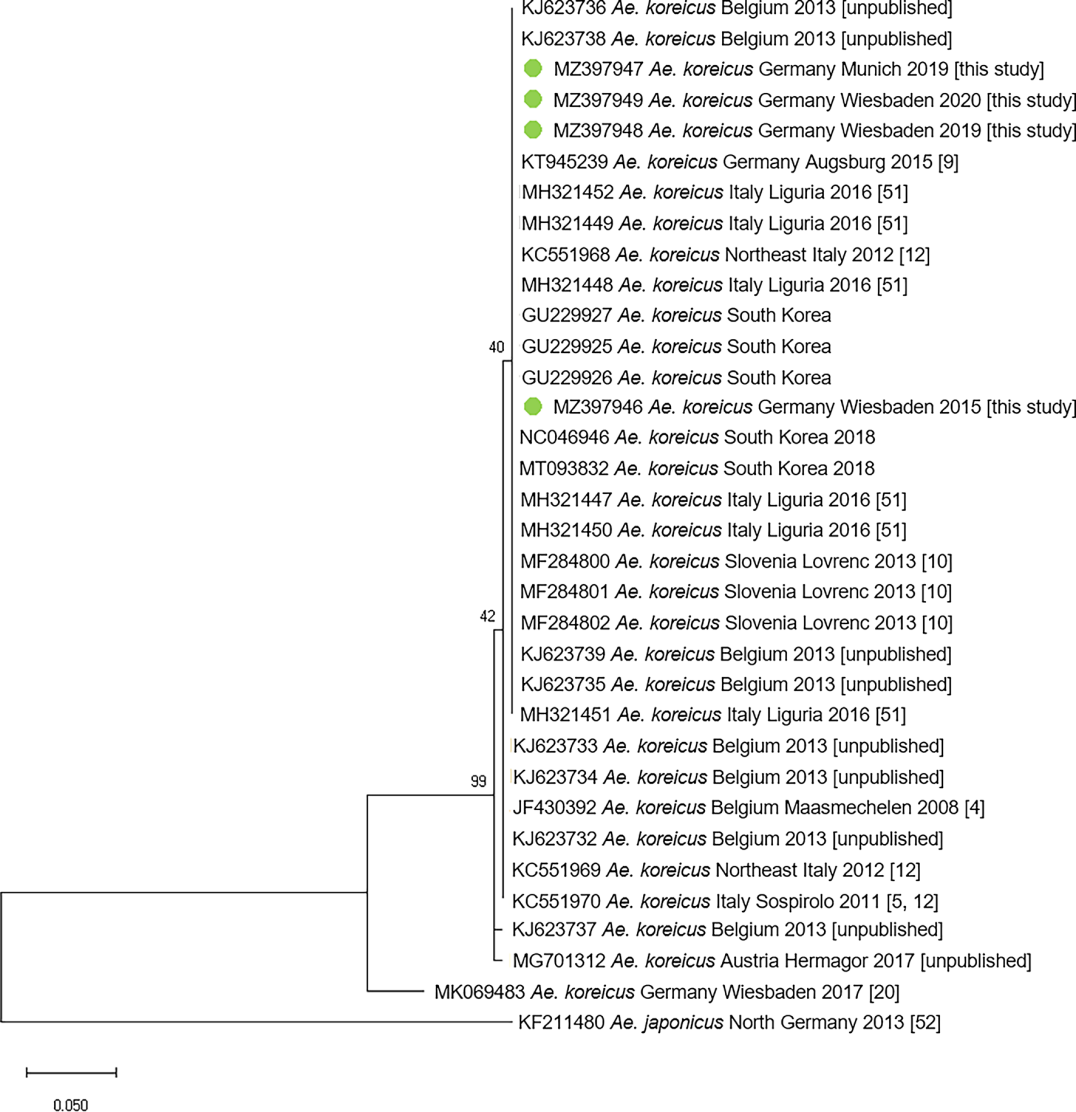
Phylogenetic tree based on partial ND4 DNA sequences of *Ae. koreicus* from this study and from GenBank. As long as available, GenBank accession numbers are supplemented by country, locality/region and year of sample collection as well as by the reference publication

ND4 sequences generated from four *Ae. koreicus* specimens collected in this study (one each collected in Wiesbaden 2015, 2019 and 2020, one collected in Munich 2019) were deposited in GenBank under the accession numbers MZ397946–MZ397949. These were phylogenetically analysed against corresponding *Ae. koreicus* sequences found in GenBank: nine from Belgium, nine from Italy, five from South Korea, three from Slovenia, one from Austria and two from previous studies in Germany. The resulting phylogenetic tree, based on DNA fragments trimmed to a consistent length of 190 bp, suggests a particularly close genetic relationship of almost all specimens, with only minor differences even between individuals from Asia and Europe (Fig. 3).

## Discussion

Tertiary to *Ae. albopictus* and *Ae. japonicus, Ae. koreicus* is another invasive mosquito vector species establishing populations on the European continent. After the detection of a population in the German municipality of Wiesbaden in 2016 [20, 26, 27], the present study could demonstrate that the species continued to occur in 2019 and 2020 in the delineated area where aquatic stages were identified in several cemeteries in addition to those previously found positive [20, 26]. Three of the new positive sampling sites (northern and northeastern Wiesbaden) represent previously unsampled cemeteries while another four, including two previously negative (northeastern and southern Wiesbaden) and two previously unsampled ones (eastern and southeastern Wiesbaden), were located slightly beyond the borders of the population area described by Pfitzner et al. [20, 26], indicating a marginal expansion of the *Ae. koreicus* population after 2018. In addition, one Mueckenatlas submission each from western (Klarenthal) and southeastern Wiesbaden (Nordenstadt) (Fig. 1b) were identified as *Ae. koreicus*, suggesting that the species had advanced into these areas, although presence could not be confirmed by cemetery inspections. However, four out of nine *Ae. koreicus* Mueckenatlas submissions were received from a settlement with an *Ae. koreicus* cemetery record in 2019 from the northeast of the Wiesbaden municipality, close to the sites with the earliest *Ae. koreicus* findings in 2015 and 2016 (Fig. 1b).

No indications exist so far on where the Wiesbaden population originates from and how introduction had taken place. Since invasive mosquitoes are sometimes imported by international air traffic [40], it was hypothesised that the initial introduction might have been through Frankfurt International Airport [20], which is only some 20 km away. However, cemeteries of villages checked between Wiesbaden and Frankfurt International Airport did not produce any *Ae. koreicus* specimens. Hence, importation of larvae or eggs through ground transport, for example of gardening equipment, as seen for *Ae. japonicus* [14], from already colonised European areas (e.g. in Belgium or Italy) is at least similarly likely. In fact, *Ae. koreicus* eggs were repeatedly collected in Switzerland by oviposition traps on a service station along a major motorway connecting Italy and Germany as well as on a railway station [41], while eggs of this species were also found in an Austrian city close to a junction of major traffic axes connected to the city by various exits [11]. More in-depth population genetic studies might provide more substantiated clues as to the origin and migration routes of *Ae. koreicus*.

### Re-examination of archived mosquito samples and examination of current Mueckenatlas submissions

After the first discovery of *Ae. koreicus* in Slovenia, individuals previously identified morphologically as *Ae. japonicus* were re-inspected and found to be mixed with *Ae. koreicus* [10]. Because *Ae. japonicus* had also been present in Germany for years, revealing a high expansion tendency, a re-analysis of “*Ae. japonicus*” stock material for admixture by *Ae. koreicus* seemed also appropriate here. In fact, archived submissions to the citizen science project Mueckenatlas from the years 2015, 2017 and 2018 turned out to be *Ae. koreicus* by morphological and confirmatory genetic identification, suggesting that the Wiesbaden population had emerged no later than 2015. In addition, *Ae. koreicus* specimens were submitted during the study period 2019/2020. These findings once more demonstrate the suitability of the Mueckenatlas to detect and track invasive mosquito species [42, 43], but also emphasise the necessity in this global world to expect, and thoroughly check for, new alien species closely related to already established invasive ones. Except for the Wiesbaden location, *Ae. koreicus*, however, could not be found among any re-inspected archived Asian bush mosquito collection material, suggesting the Wiesbaden population to be the only one in Germany. According to a field survey, also the Mueckenatlas submissions from Munich in 2019 must be considered single individual importations not connected to a resident population.

### Expansion behaviour

Even though two species as closely related and as similar in appearance as *Ae. japonicus* and *Ae. koreicus* can be found in the same area, they are not necessarily characterised by the same ecological requirements and may exhibit different behaviours. It is unclear exactly which factors are responsible for the species’ ability to become established and spread. Both species originate from temperate areas in Asia [2] and can therefore be expected to be adapted to Central European climatic conditions. In fact, eggs of both species are able to overwinter in Europe, so that populations once established have remained present through the years. However, in contrast to *Ae. japonicus*, which generally exhibits a considerable active and passive dispersal rate in Europe, although stationary populations have been observed [24, 25], European populations of *Ae. koreicus* have mainly remained quite stationary and restricted to the area of initial establishment [44, 45]. Thus, the observed negligible spread of the German population fits well with studies on other European *Ae. koreicus* populations. The population in Belgium, which has been observed from 2008 onwards through the years, has remained restricted to an area even smaller than the German one [44, 46]. Contrasting other European populations, however, the current Italian *Ae. koreicus* population has a large distribution area with noticeable spread into various parts of northern Italy up to the border with Switzerland [12]. Due to various morphological similarities, the Belgian and Italian *Ae. koreicus* populations are associated with an origin on Jeju-do Island, Korea [4, 5], where one of two Korean bush mosquito variants can be found [2]. By contrast, the German population is morphologically more similar to the population variant observed on the Korean mainland [20]. Hitherto, there has been no evidence that the *Ae. koreicus* variants differ in their environmental requirements or behaviour that may influence expansion dynamics. Whether the lack of expansiveness is due to ecological conditions not met in Europe or just to the specific invasive mosquito strain(s) and their genetic make-up therefore remains a question for further studies. ND4 sequence data, at least as far as the trimmed fragment available for comparison from GenBank entries is concerned, do not appear to be informative enough to deduce genetic relationships and origins, and thus to conclude on displacement and migration routes, of geographic populations.

### Primer evaluation

CO1-barcoding, based on PCR with LCO1490 and HCO2198 primers according to Hébert et al. [36] and Folmer et al. [37], has been shown successful in identifying many mosquito species worldwide (e.g. [47–49]). Thus, this PCR approach has been used for routine identification of mosquitoes collected in Germany for many years [50], including attempts to genetically verify morphologically pre-identified *Ae. koreicus* specimens in this study. However, the above primers obviously did not anneal correctly which could be attributed to base pair mismatching with the rDNA of this specific mosquito species. By contrast, three other primer pairs, MTFN/MTRN, PanCuli-COX1-211F/PanCuli-COX1-727R and N4J-8502/ND4R1X [31, 32, 37], targeting CO1 and ND4 genetic markers, respectively, were successfully used for molecular identification of *Ae. koreicus*. A slight modification of the LCO1490/HCO2198 nucleotide sequences solved the annealing problem but prevented functionality with other mosquito species, while an equal mixture of original and modified primers worked well with *Ae. koreicus* and other mosquito species tested. Accordingly, studies applying genetic identification of mosquitoes from an area in which *Ae. koreicus* occurs, but not specifically focusing on that species, should well consider which genetic marker and primer pairs to use in order to obtain unambiguous identification results and to minimise work at the same time.

## Conclusions

Based on the documentation of *Ae. koreicus* sampling sites from 2016 to 2018 and the finding of this species at new sites in the Wiesbaden area in 2019 and 2020, it appears that the only currently known German population of *Ae. koreicus* is more or less stable five years after its establishment. Apparently, an expansion, if it has taken place, is negligible. However, the present evaluation was based on cemetery inspections alone, as cemeteries simultaneously offer plenty of potential mosquito breeding sites and unrestricted access to investigators [29]. Including allotment and private gardens might well show a more widespread distribution of *Ae. koreicus* in the Wiesbaden area than detected in this study. Spreading capacity as well as origin and introduction route(s) of *Ae. koreicus* are major topics that need to be addressed in more detail in the future. The demonstrated establishment and occasional introduction of this potential mosquito vector species in Germany, however, should be reason enough to continue mosquito monitoring, paying special attention to the distinction between *Ae. koreicus* and *Ae. japonicus*.

## Data Availability

Data supporting the conclusions of this article are included within the article.
